# Knowledge of Cervical Cancer and Human Papillomavirus among Japanese Women

**DOI:** 10.31557/APJCP.2020.21.12.3527

**Published:** 2020-12

**Authors:** Yuki Nakao, Ai Sasaki, Taku Obara, Shinya Abe, Kaori Furusaki, Mari Higaki, Shouko Yoshimachi, Teruaki Gotou

**Affiliations:** 1 *Tsuruha Pharmacies Co., Ltd. Sapporo, Hokkaido, Japan. *; 2 *Division of Preventive Medicine and Epidemiology, Tohoku University Tohoku Medical Megabank Organization, Sendai, Japan. *; 3 *Department of Molecular and Epidemiology, Tohoku University Graduate School of Medicine, Sendai, Miyagi, Japan. *; 4 *Department of Pharmaceutical Sciences, Tohoku University Hospital, Sendai, Miyagi, Japan. *; 5 *Tsuruha Holdings, Sapporo, Hokkaido, Japan. *

**Keywords:** Knowledge, cervical cancer, human papillomavirus, human papillomavirus vaccine, cervical cancer test

## Abstract

**Background:**

The combination of human papillomavirus (HPV) vaccination and cervical cancer tests are globally recommended. Although knowledge regarding cervical cancer and HPV and experience of HPV vaccination are reportedly closely associated, the associations between knowledge and frequency of cervical cancer testing are unclear.

**Methods:**

We conducted a questionnaire survey regarding the knowledge of cervical cancer and HPV and experience of HPV vaccination and frequency of cervical cancer testing including cervical cytology and HPV testing.

**Results:**

Among 99 women who visited Tsuruha Festa, most of the 77 non-medical workers who received information on cervical cancer and HPV through the Internet were not more likely to have knowledge about cervical cancer and HPV than were 12 medical workers who had gotten information in vocational school or university curriculum. The rates of HPV vaccination, cervical cytology, and HPV testing were 4.0%, 14.1%, and 4.0%, respectively, among participants and did not differ significantly according to participant job and age. Knowledge about cervical cancer and HPV was associated with experience of HPV vaccination and frequency of cervical cytology and was not associated with frequency of HPV testing.

**Conclusions:**

We observed insufficient knowledge about cervical cancer and HPV among non-medical workers as well as low HPV vaccination, cervical cytology, and HPV testing rates, and knowledge about cervical cancer and HPV to which frequency of cervical cancer testing were partially related. Therefore, the government should take measures to enhance public awareness about cervical cancer and HPV through social media such as the Internet.

## Introduction

Cervical cancer was the fourth most common cancer in women with an estimated 570,000 new cases in 2018, representing 6.6% of all cancer in women and 311,000 deaths (Arbyn et al., 2020). In Japan, cervical cancer affects about 10,000 and kills about 2,800 women annually. The numbers of both patients and deaths have been gradually increasing since the 1990s (Bruni et al., 2019). In particular, the incidence is higher in the younger generation (aged between 20 and 40 years), compared to those of other age groups. 

Long-lasting human papillomavirus (HPV) infection is a main factor contributing to the development of cervical cancer. The HPV vaccine can prevent infection with HPV types 16 and 18, two high-risk HPVs that cause about 70% of cervical cancers. In Japan, funding for HPV vaccination began in 2010 for girls aged 12–16 years, with three-dose coverage initially reaching more than 70%. In June 2013, 2 months after its formal inclusion in the Japanese national immunization program, proactive recommendations for HPV vaccination were suspended. HPV vaccine coverage subsequently dropped to less than 1% and has remained at this level (Simms et al., 2020). The proactive recommendation of HPV vaccination remains controversial.

Worldwide, the screening modalities for cervical cancer include cervical cytology (Pap test), visual inspection, and HPV deoxyribonucleic acid (DNA) testing (HPV test). The HPV test can be used in combination with cervical cytology to screen for cervical cancer and it is firstly recommended in primary cervical cancer screening (Jeronimo et al., 2016). In Japan, cervical cytology is performed for primary screening for cervical cancer (Hamashima et al., 2010). When used with high-risk HPV polymerase chain reaction assays, testing on self-samples was similarly accurate as that for clinician samples (Arbyn et al., 2018). Therefore, HPV tests can be performed with both clinician and self-sampling.

Questionnaire surveys on the knowledge of cervical cancer and HPV vaccination coverage have increased in recent years. A meta-analysis of 58 studies, as well as systematic reviews, nationwide interview surveys, and population-based surveys on cervical cancer have been reported (Arrossi et al., 2012; Oh et al., 2010; Taebi et al., 2019; Zhang et al., 2016). In Japan, only one study has assessed knowledge regarding cervical cancer and HPV and willingness to receive HPV vaccination (Suzuki et al., 2019). The study reported on willingness to receive HPV vaccination but did not describe the actual experience of HPV vaccination, cervical cytology, and HPV testing. The World Health Organization Europe indicates that the combination of HPV vaccination and regular screening is the most effective way for women to protect against cervical cancer (World Health Organization Europe, 2017). Therefore, we aimed to investigate the association between knowledge about cervical cancer and HPV and experience of HPV vaccination and frequency of cervical cancer testing including cervical cytology and HPV tests.

## Materials and Methods

We provided self-collected HPV tests and also conducted a questionnaire survey regarding cervical cancer and HPV on May 18 and 19 and August 24 and 25, 2019, at Tsuruha Festa as part of 2-day general public events related to health and beauty provided by Tsuruha Pharmacies Co., Ltd., a drugstore chain company in Japan. We asked women aged 20–39 years visiting these events to participate in this survey. A total of 99 women participated in the survey. We asked participants to answer the questionnaire and collected them immediately after completion. The questionnaire comprised items relating to the participants’ present knowledge of cervical cancer and HPV as well as their experiences with HPV vaccination and cervical cancer tests including cell cytology and HPV tests (Appendix). After completing the questionnaires, we provided the correct answers and relevant knowledge about HPV and the vaccine. Ethical approval for the study was obtained from the Institutional Review Board of Tsuruha Pharmacies Co., Ltd.

Women who responded “I understand” to the question regarding their understanding of cervical cancer and HPV were classified into the “yes” group, while those who answered “I could not understand, but I have heard” or “I did not know” were classified into the “no” group. Women in the “yes” group were considered to have knowledge about the topic of each question on cervical cancer and HPV. We compared the proportions of women with such knowledge according to their job (medical or non-medical workers) and age (20–29 years or 30–39 years). Among women with knowledge about cervical cancer and HPV, we also compared the sources of information between medical and non-medical workers. We also compared experience of HPV vaccination and frequency of cervical cancer testing including cervical cytology and HPV testing according to their jobs, ages, and knowledge about cervical cancer and HPV. Fishers’ exact and Cochran-Armitage trend tests were conducted, as appropriate. All statistical analyses were conducted using SAS version 9.4 (SAS Institute Inc., Cary, NC, USA).

## Results

**Figure 1:**
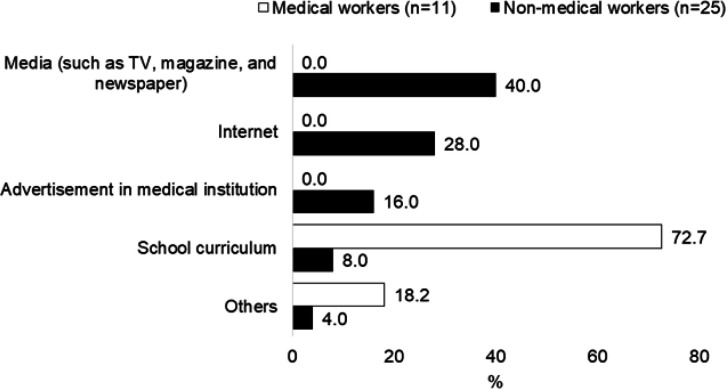
Source of Information on Cervical Cancer

Among 99 women, 75 were non-medical workers and 27 were aged 20–29 years. Knowledge about cervical cancer and HPV according to job and age are shown in Table 1. Non-medical workers were not more likely to have knowledge about cervical cancer, HPV, HPV vaccine, and cervical cancer testing than medical workers. No significant differences were observed in the knowledge of government and company subsidies between non-medical and medical workers. Similarly, no significant differences in any knowledge were observed between age groups. The sources of information on cervical cancer according to the participants’ jobs are shown in Figure 1. Forty percent of non-medical workers got information on cervical cancer and HPV from the Internet, followed by the TV (28%), advertisements in medical institutions (16%), and educational curriculum (6%). Most medical workers received information on cervical cancer and HPV from their school curriculum (72%).

Experience of HPV vaccination according to participants’ job, age, and knowledge are shown in Table 2. The rate of HPV vaccination was 4.0% among the 99 women. No significant differences in experience of HPV vaccination were observed between non-medical and medical workers. Women aged 20–29 years were more likely to receive the HPV vaccine compared to those aged 30–39 years. Participants with knowledge of cervical cancer, the relationship between HPV and cervical cancer, the HPV vaccine, and cytology were significantly more likely to receive HPV vaccination than were those without such knowledge. The HPV vaccine coverage was 15.8% in those with knowledge about the HPV vaccine and 1.3% in those without knowledge. No significant differences in vaccination rates were observed between participants with or without knowledge regarding the subsidization of cervical cancer and HPV tests by governments or companies.

The frequency of cervical cytology according to participants’ job, age, and knowledge are shown in Table 3. The rates of cervical cytology once every 2 years and once every 2 years or irregularly were 14.1% and 62.6%, respectively, in the 99 women. No significant differences were observed in frequency of cervical cytology once every 2 years between non-medical and medical workers. Women aged 30–39 years were more likely to have cervical cytology once every 2 years compared to those aged 20–29 years. Knowledge about the development of cervical cancer and subsidization of cervical cancer testing by companies or governments were associated with regular and irregular cervical cytology. The rate of regular cytology was around 30% among women with knowledge and less than 10% in those without knowledge. The rate of experience with cervical cytology once every 2 years or irregularly was over 60%, regardless of the level of knowledge of cervical cytology. 

The frequency of HPV testing according to participants’ job, age, and knowledge are shown in Table 4. The rates of HPV testing once every 2 years and once every 2 years or irregularly were 4.0% and 29.3%, respectively, in the 99 women. No significant differences in frequency of HPV testing were observed regardless of participants’ job and age. The rate of HPV testing once every 2 years was less than 10% while that for once every 2 years or irregularly was around 30%. No significant differences in HPV testing rates were observed regardless of participants’ knowledge. 

**Table 1:** Knowledge Related to Cervical Cancer and HPV

		Job			Age		
		"Medical workers" n=14	"Non-medicalworkers" n=76	P*	30-39 years old n=27	20-29 years old n=67	P*
Do you know about cervical cancer which develops in uterine cervix?							
	Yes	78.6	32.9	0.002	44.4	37.3	0.642
Do you know about HPV with which most people experience infection?							
	Yes	57.1	17.1	0.003	25.9	20.9	0.595
Do you know that persistent infection with HPV can lead to cervical cancer?							
	Yes	64.3	15.8	0.000	18.5	26.9	0.441
Do you know that HPV can be transmitted by sexual intercourse?							
	Yes	71.4	35.5	0.018	40.7	41.8	1.000
Do you know about HPV vaccines?							
	Yes	57.1	14.5	0.001	18.5	17.9	1.000
Do you know about cervical cytology?							
	Yes	50.0	25.0	0.104	29.6	28.4	1.000
Do you know about HPV testing?							
	Yes	92.9	75.0	0.179	70.4	80.6	0.288
Do you know differences of the cervical cytology and the HPV testing?							
	Yes	35.7	5.3	0.004	14.8	7.5	0.273
Do you know that you can receive cervical cancer tests at a subsidized price by companies or governments?							
	Yes	50.0	46.1	1.000	40.7	47.8	0.649

HPV, human papillomavirus; *fishers' exact test.

**Table 2:** Attitude toward HPV Vaccination

	%	P
Job		
Non-medical workers	2.6	0.113
Medical workers	14.3	
Age		
30-39 years old	0.0	0.022
20-29 years old	11.1	
Do you know about cervical cancer which develops in uterine cervix?		
No	0.0	0.020
Yes	10.5	
Do you know about HPV with which most people experience infection?		
No	1.3	0.033
Yes	13.6	
Do you know that persistent infection with HPV can lead to cervical cancer?		
No	1.4	0.049
Yes	12.0	
Do you know that HPV can be transmitted by sexual intercourse?		
No	1.7	0.304
Yes	7.3	
Do you know about HPV vaccines?		
No	1.3	0.022
Yes	15.8	
Do you know about cervical cytology?		
No	0.0	0.005
Yes	14.3	
Do you know about HPV testing?		
No	4.4	1.000
Yes	4.0	
Do you know differences of the cervical cytology and the HPV testing?		
No	2.2	0.050
Yes	20.0	
Do you know that you can receive cervical cancer tests at a subsidized price by companies or governments?		
No	3.6	1.000
Yes	4.5	

HPV, human papillomavirus; *fishers' exact test.

**Table 3:** Attitude toward Cervical Cytology

	Once every two years, %	P	"Once every two years or irregular, %"	P
Job				
Non-medical workers	21.4	0.451	64.3	1.000
Medical workers	14.5		61.8	
Age				
30-39 years old	16.4	0.335	71.6	0.018
20-29 years old	7.4		44.4	
Do you know about cervical cancer which develops in uterine cervix?				
No	4.9	0.002	52.5	0.010
Yes	28.9		79.0	
Do you know about HPV with which most people experience infection?				
No	9.1	0.013	61.0	0.623
Yes	31.8		68.2	
Do you know that persistent infection with HPV can lead to cervical cancer?				
No	9.5	0.041	60.8	0.635
Yes	28.0		68.0	
Do you know that HPV can be transmitted by sexual intercourse?				
No	10.3	0.246	65.5	0.531
Yes	19.5		58.5	
Do you know about HPV vaccines?				
No	8.8	0.005	62.5	1.000
Yes	36.8		63.2	
Do you know about cervical cytology?				
No	11.3	0.211	63.4	0.821
Yes	21.4		60.7	
Do you know about HPV testing?				
No	0.0	0.035	39.1	0.013
Yes	18.4		69.7	
Do you know differences of the cervical cytology and the HPV testing?				
No	11.2	0.033	62.9	1.000
Yes	40.0		60.0	
Do you know that you can receive cervical cancer tests at a subsidized price by companies or governments?				
No	3.6	0.001	47.3	0.001
Yes	27.3		81.8	

**Table 4:** Attitude toward HPV Testing

	Once every two years, %	P	"Once every two years or irregular, %"	P
Job				
Non-medical workers	5.3	1.000	30.3	1.000
Medical workers	0.0		28.6	
Age				
30-39 years old	3.0	1.000	32.8	0.455
20-29 years old	3.7		22.2	
Do you know about cervical cancer which develops in uterine cervix?				
No, %	3.3	0.637	26.2	0.497
Yes, %	5.3		34.2	
Do you know about HPV with which most people experience infection?				
No, %	2.6	0.213	24.7	0.069
Yes, %	9.1		45.5	
Do you know that persistent infection with HPV can lead to cervical cancer?				
No, %	2.7	0.264	27.0	0.449
Yes, %	8.0		36.0	
Do you know that HPV can be transmitted by sexual intercourse?				
No, %	3.4	1.000	27.6	0.662
Yes, %	4.9		31.7	
Do you know about HPV vaccines?				
No, %	3.8	0.580	28.8	0.786
Yes, %	5.3		31.6	
Do you know about cervical cytology?				
No, %	4.2	1.000	28.2	0.807
Yes, %	3.6		32.1	
Do you know about HPV testing?				
No, %	0.0	0.571	26.1	0.798
Yes, %	5.3		30.3	
Do you know differences of the cervical cytology and the HPV testing?				
No, %	3.4	0.351	25.8	0.060
Yes, %	10.0		60.0	
Do you know that you can receive cervical cancer tests at a subsidized price by companies or governments?				
No, %	1.8	0.320	21.8	0.079
Yes, %	6.8		38.6	

HPV, human papillomavirus; *fishers' exact test.

## Discussion

We found that non-medical workers did not have sufficient perceptions about cervical cancer and HPV, that social systems including the government’s financial support for cervical cancer testing were not sufficiently recognized even among medical workers, and that knowledge related to cervical cancer and HPV was associated with experience of HPV vaccination and frequency of cervical cancer testing.

Medical workers received information on cervical cancer and HPV as part of their school curriculum, while non-medical workers received such information from the Internet, TV, and other media. The media may be an important tool to provide non-medical workers knowledge of cervical cancer and HPV. Other countries have used the Internet and social media to disseminate health-related information (Bianco et al., 2013; Liu et al., 2020; Zucco et al., 2018). Therefore, the Japanese government should consider social media in their communication strategies to promote appropriate Web use for the dissemination of medical information on cervical cancer itself to non-medical workers and social systems including the government’s financial support for cervical cancer testing to both non-medical and medical workers.

In our study, participants aged 20–29 years with knowledge related to cervical cancer, HPV, and HPV vaccine were significantly more likely to receive HPV vaccination than those without this knowledge. Although the number of participants in the present study was small and the conclusions correspondingly limited, the proactive recommendation for HPV vaccination as was performed until 2013 in Japan may have a great impact on rate of HPV vaccination among participants aged 20–29 years. High levels of knowledge about HPV and the vaccine are related to a greater willingness to receive the vaccine. Studies among undergraduate students reported that the level of knowledge about HPV and HPV vaccine and health education were important predictors of increased vaccination rates (Liu et al., 2020; Navalpakam et al., 2016; Oz et al., 2018) and the main reasons for rejecting the HPV vaccination were insufficient information about the vaccine and possible unknown side effects (Oz et al., 2018). A previous study reported that, after educational instruction consisting of a 1-hour group lecture followed by the same questionnaire as that provided after informed consent, vaccine acceptability increased significantly from 77% to 90% among employed women (Chang et al., 2013). Therefore, informative group lectures may be effective for the improvement of HPV-related knowledge and HPV vaccine acceptability. However, as the HPV vaccination rate was low even among participants in our study with knowledge about HPV vaccination, the provision of information and education including not only the need for HPV vaccination but also correct knowledge about adverse reactions after vaccination is necessary.

In our study, the rates of experience with cervical cytology once every 2 years was similar to the rate of cervical cancer testing within 1 year among women aged 20–49 years in Japan (<30%) (Bruni et al., 2019). Participants aged 30–39 years with knowledge related to cervical cancer, HPV, and HPV vaccine were significantly more likely to have experienced cervical cytology and no differences were observed in the rates of experience with cervical cytology between participants regardless of their knowledge about cervical cytology. In Japan, cervical cancer screening programs with cervical cytology are conducted once every 2 years and are actively recommended for people over 20 years of age. Therefore, participants with knowledge related to cervical cancer might be willing to receive the screening, resulting in their having experienced cervical cytology. This program might also explain the rate of experience with cervical cytology once every 2 years or irregularly over 60%, regardless of the level of knowledge about cervical cytology in this study. In contrast, the rate of experience with HPV testing was associated with knowledge about HPV testing. One explanation may be that it is necessary to actively acquire to receive an HPV test in Japan (Simms et al., 2020). Respondents to this questionnaire survey were participants in self-sampling HPV tests at Tsuruha Festa and, while many had knowledge of HPV testing, the implementation rate was low. The general population may have less knowledge and fewer experiences with HPV testing compared to those in our study participants. Thus, increasing awareness of HPV testing and creating an environment that facilitates HPV testing are required. 

In Conclusion, the results of this study demonstrated an insufficient knowledge of cervical cancer and HPV including HPV vaccinations and cervical cancer tests in non-medical workers. The HPV vaccination, cervical cytology, and HPV testing rates were very low, and were partially associated with the rates of receiving HPV vaccinations and cervical cancer tests. Our findings suggested that knowledge of cervical cancer and HPV and national programs such as active recommendations to receive HPV vaccination and cervical cancer tests would affect the health behaviors related to cervical cancer, HPV, HPV vaccine, and cervical cancer testing.
